# A New Insight into the Threshold and Oscillatory Regimes in Plant-Pathogen Models: A Nutrient-Driven Approach

**DOI:** 10.1007/s11538-026-01682-8

**Published:** 2026-06-15

**Authors:** Dhruba Pariyar Damay, Angela Peace

**Affiliations:** 1 Department of Mathematics and Statistics, Texas Tech University, Lubbock, TX USA; 2 Department of Mathematics, Virginia Tech, Blacksburg, VA USA; 3 Virginia Tech Center for the Mathematics of Biosystems, Blacksburg, VA USA

**Keywords:** Plant-pathogen, Ecosystem disease, Nutrient cycling

## Abstract

Across ecosystems, autotroph growth and susceptibility to disease are strongly constrained by the availability of essential nutrients such as nitrogen and phosphorus. Understanding how nutrient availability influences disease transmission is important for predicting disease persistence, outbreak risk, and long-term ecosystem dynamics under changing environmental conditions. At the same time, infectious diseases in autotrophs can reshape ecosystem processes by altering elemental recycling and the nutrient supply available to hosts. Here, we formulate a five-dimensional deterministic system of nonlinear ordinary differential equations within a disease-mediated nutrient dynamic framework. We incorporate novel nutrient-driven transmission and nonlinear resource uptake kinetics to capture the bidirectional relationships linking elemental cycles with infectious disease in a natural forest ecosystem. Using a combination of qualitative mathematical analysis, including proofs of solutions boundedness and derivations of basic reproductive number, and numerical bifurcation analyses, we evaluate the system’s long-term behavior. Our results show that nutrient-disease feedbacks strongly regulate the distribution of host densities and nutrients between autotrophs and the abiotic environment. Incorporating nutrient-driven transmission reveals bifurcation structures distinct from frameworks with constant transmission, highlighting high sensitivity to nutrient availability and stronger nonlinear feedbacks. Bifurcation analyses indicate that nutrient enrichment lowers the transmission threshold for disease persistence and accelerates the onset of oscillatory dynamics with greater amplitude under high nutrient levels. Similarly, higher transmission rates reduce the nutrient threshold for disease persistence and shift oscillatory dynamics to emerge at lower nutrient levels. We further show that even small differences in infected host uptake rates strongly influence dynamics: lower uptake dampens oscillations and weakens feedbacks, whereas higher uptake amplifies bottom-up nutrient effects on disease and reinforces top-down effects on nutrient cycling, producing pronounced limit cycles in hosts, nutrients, and prevalence. Overall, nutrient-driven transmission alters thresholds and oscillatory regimes in ecosystem disease models, leading to dynamics that are not captured under constant transmission assumptions. This work advances applied ecosystem and ecological disease sciences by improving our understanding of disease transmission processes in plant communities.

## Introduction

Nutrients are essential not only for plant growth and development but also for shaping disease resistance and control (Datnoff et al. 2007). Although resistance and tolerance are genetically determined (Agrios 2005), they are strongly influenced by environmental factors, especially nutrient deficiencies, toxicities, and stresses such as drought, extreme temperatures, or excessive sunlight, all of which weaken plants and increase their vulnerability to pathogens (Palti 1981; Marschner 1995; Krauss 1999). In contrast, a balanced nutrient supply can buffer these stresses, lowering disease severity and strengthening plant resilience (Dordas 2008). In addition, micronutrients are critical for supporting plant physiological and biochemical defenses, with deficiencies often associated with greater disease severity (Marschner 1995; Bolle-Jones and Hilton 1956; Schutte 1967). On the other hand, in some scenarios, high nutrient levels have been shown to increase disease prevalence through density-dependent transmission and direct benefits to pathogen growth demands (Van de Waal et al. 2023). Together, these findings highlight nutrient availability as a central factor linking plant health and disease outcomes and establish the basis of this investigation.

Environmental conditions and host physiological status play important roles in disease emergence, with nutrient availability and environmental stress influencing host susceptibility, pathogen pressure, and disease dynamics across ecosystems (Cobb and Metz 2017). Forest diseases can have substantial ecological and economic consequences by affecting tree health, forest productivity, biodiversity, and the ecosystem services that forests provide. These impacts underscore the importance of understanding the mechanisms that govern disease transmission, persistence, and spread. In many forest systems, root diseases are particularly challenging because they operate largely belowground, where transmission pathways are difficult to observe directly (Goheen and Otrosina 1999). This hidden nature complicates diagnosis and management efforts and underscores the importance of improving our understanding of disease transmission processes in plant communities.

Shifts in the availability of carbon (C), nitrogen (N), and phosphorus (P) strongly influence biological processes, from cellular function to community dynamics and food-web interactions (Cherif et al. 2017; Sterner and Elser 2002; Waal et al. 2010). Changes in nutrient supply, including those driven by anthropogenic enrichment, can alter disease dynamics by affecting pathogen transmission, host physiology, and vector abundance (Borer et al. 2016; Preston et al. 2016), with these shifts feeding back to ecosystem processes such as primary productivity, nutrient uptake, and recycling. Because pathogens act as consumers constrained by the energy and nutrients of their hosts, they can modify host physiology, defenses, and ecological functioning (Smith 2007; Hatcher et al. 2012). Across both managed and natural systems, nutrient availability plays a central role in shaping disease outcomes and mediating host-pathogen interactions, with cascading consequences for community structure and ecosystem functioning (Dordas 2008).

Because pathogens are widespread, understanding the functional links between disease and nutrient dynamics has the potential to advance both disease ecology and ecosystem ecology (Borer et al. 2021). Although these fields are inherently connected, they have developed along separate trajectories-one centered on host population dynamics (Anderson and May 1979), the other on nutrient fluxes and pools (Chapin et al. 2011). This disciplinary divide has limited progress in understanding how disease and ecosystem processes interact (Ostfeld et al. 2008; Preston et al. 2016). The coevolution of hosts and pathogens under changing environments adds further complexity to ecosystem disease dynamics (Chakraborty and Datta 2003), underscoring the need for integrative approaches. Research in natural systems has proven especially valuable for bridging this gap, showing how nutrient influx and recycling can strongly influence carbon pools as well as the prevalence and spread of infectious disease (Borer et al. 2021). Together, these perspectives motivated the disease-mediated nutrient dynamics (DND) framework developed by Borer et al. (2022), which demonstrates that feedbacks between nutrient cycling and infectious disease can generate complex and potentially destabilizing dynamics in host populations, infection prevalence, and nutrient pools. This framework provides a mechanistic basis for simultaneously examining nutrient and disease dynamics within a unified ecological model.

When nutrients are limited, plants often respond by modifying their root architecture to improve nutrient uptake. They may produce more fine roots, increase lateral root growth, and expand their root systems to explore a larger volume of soil in search of scarce resources (Gojon et al. 2009; Hinsinger et al. 2009; Gruber et al. 2013). These adaptive changes increase interactions between roots and the surrounding soil and can also lead to greater overlap among neighboring plant root systems competing for the same nutrient patches (Fitter et al. 2002; Giehl et al. 2014). As root systems become more interconnected, the number of shared soil pathways and root-to-root contact points may also increase, creating favorable conditions for the transmission of soil-borne pathogens. Thus, nutrient limitation can enhance conditions favorable for pathogen transmission within plant communities by increasing root proliferation and spatial overlap. These responses may also involve tradeoffs between resource acquisition and disease risk. Root traits such as extensive branching and high specific root length have been associated with greater susceptibility to soil-borne pathogens and herbivores (Maherali 2014; Laliberté et al. 2015; McCormack et al. 2015; Weemstra et al. 2019). Together, these observations suggest that the same strategies plants use to cope with nutrient limitation may also enhance conditions favorable for pathogen transmission.

Disease and ecosystem nutrient dynamics are intricately linked through multiple pathways. Early studies often treated these relationships as unidirectional, focusing either on the effects of nutrients on disease (Aalto et al. 2015; Borer et al. 2016; Civitello et al. 2018; Dordas 2008) or on how infection alters nutrient dynamics (Eviner and Likens 2008; Fischhoff et al. 2020; Preston et al. 2016). In reality, pathogens and nutrient supply jointly influence host stoichiometry, growth, and survival, with cascading effects on nutrient cycling and community structure (Borer et al. 2021, 2022; Vannatta and Minchella 2018). More recent perspectives emphasize the inherently bidirectional nature of these interactions, highlighting feedbacks that can generate complex and emergent dynamics (Borer et al. 2021, 2022; Seabloom et al. 2023).

Recent studies have increasingly emphasized the close connections between nutrient availability and infectious disease dynamics. By integrating concepts from disease ecology and ecosystem ecology, Borer et al. (2021) showed that nutrient supply and recycling can influence both disease prevalence and ecosystem processes, highlighting the importance of considering these feedbacks within a unified framework. This perspective was further developed through the disease-mediated nutrient dynamics (DND) framework, which demonstrated that reciprocal interactions between nutrient cycling and infectious disease can generate complex ecological dynamics (Borer et al. 2022). More recently, Van de Waal et al. (2023) showed that nutrient availability can affect pathogen transmission through its influence on host immunity and pathogen infectivity, leading to disease outcomes that differ substantially from those predicted by models with fixed transmission rates. Together, these studies highlight the growing recognition that nutrient-mediated processes play a fundamental role in disease dynamics and motivate further investigation of how nutrient-dependent transmission influences disease persistence, thresholds, and long-term system behavior. While these studies had thoroughly advanced the field, a major limitation is that they treat pathogen transmission rates as constant or decoupled from the explicit, environmental nutrient pools.

Previously developed models explicitly couple pathogens with ecosystem nutrient flows by linking nutrient availability to the growth of susceptible and infected autotroph hosts and incorporating nutrient recycling through host mortality, thereby connecting disease ecology with ecosystem ecology and providing a foundation for new lines of inquiry (Borer et al. 2021, 2022). Building on this framework, we extend the model by incorporating nutrient-driven transmission in place of a constant rate to examine further how resource-disease feedbacks shape both ecosystem functioning and epidemiological outcomes. In particular, we formulate our framework as a deterministic model using a five-dimensional system of ordinary differential equations tracking host biomass, disease, and nutrient dynamics. In both frameworks, hosts are assumed to grow logistically, with growth constrained by low internal nutrient quotas under nutrient-poor conditions and by light competition when nutrients are abundant, consistent with prior formulations (Loladze et al. 2000).

Furthermore, we introduce distinct nutrient uptake functions for susceptible and infected hosts, and define a nutrient-dependent transmission function. We assume that environmental nutrient availability ($N_E$) can influence disease transmission through plant-soil interactions. Under nutrient-limited conditions, plants often respond by increasing fine-root production, lateral root growth, and root system expansion to improve nutrient acquisition (Gojon et al. 2009; Hinsinger et al. 2009; Gruber et al. 2013). These responses can increase overlap among neighboring root systems and the extent of shared soil pathways (Fitter et al. 2002; Giehl et al. 2014), potentially creating additional opportunities for the spread of soil-borne pathogens through root-to-root contact. Moreover, root traits associated with resource acquisition, such as extensive branching and high specific root length, have been linked to greater susceptibility to soil-borne pathogens and herbivores (Maherali 2014; Laliberté et al. 2015; McCormack et al. 2015; Weemstra et al. 2019). Together, these observations suggest that the same adaptations that enhance nutrient acquisition under nutrient stress may also modify conditions favorable for pathogen transmission, providing biological motivation for representing transmission as a function of environmental nutrient availability. Allowing nutrient uptake to vary with infection status reflects empirical evidence that infected and uninfected autotrophs often differ in nutrient acquisition and physiological performance (Dordas 2008; Fones and Gurr 2017). We focus on nutrient stress as a key driver, thereby enriching the feedback between disease and nutrient dynamics in our extended model.

While the underlying stoichiometric structure and growth formulation follow existing disease-nutrient frameworks (Borer et al. 2021, 2022), the key novelty of this work lies in introducing a nutrient-dependent transmission function, $\beta (N_E)$, which directly links environmental nutrient availability to pathogen transmission. This extension allows us to explore how nutrient-mediated transmission feedbacks reshape both epidemiological thresholds and ecosystem-level dynamics, generating behaviors not captured under constant transmission assumptions. To verify the mathematical and biological integrity of our novel model, we conduct qualitative mathematical analyses to prove the solutions positivity and boundedness, analytically derive the basic reproduction number $\mathcal {R}_0$, and perform numerical bifurcation analyses. Incorporating nutrient-driven transmission produces bifurcation patterns distinct from the constant transmission model, revealing strong sensitivity to nutrient availability and nonlinear feedbacks. Nutrient enrichment lowers thresholds for disease persistence and promotes earlier, higher-amplitude oscillations, while higher transmission rates shift these dynamics to lower nutrient levels. Differences in infected host uptake further shape outcomes, with higher uptake amplifying nutrient-disease feedbacks and generating pronounced cycles.

Two-parameter bifurcation analyses demonstrate that nutrient-disease dynamics are governed by thresholds in total nutrient concentration (*N*) and carbon-dependent carrying capacity (*K*), with high *N* and *K* maximizing biomass and nutrient concentrations, low values constraining disease persistence, and shifts in *N*-*K* space driving transitions between equilibria, oscillations, and extinction. These results highlight the significance of the model as a theoretical framework for linking nutrient and disease interactions in ecosystems.

The model is described in Section 2. In Section 3 we establish the positivity and boundedness of solutions to ensure biological relevance, derive the disease-free equilibria and the basic reproduction number, and analytically examine their stability properties. We also investigate the existence and local stability of endemic solutions numerically. Section 4 presents the numerical analysis of the model, including simulations, sensitivity analyses, and bifurcation analyses. Finally, conclusions and possible future explorations are discussed in Section 5.

## Model Construction

Borer et al. (2022) developed a disease-mediated nutrient dynamics model that describes how nutrient availability influences the growth of susceptible and infected autotrophic hosts and tracks the return of nutrients to the environment after host death. The model incorporates feedback loops by linking disease dynamics with elemental cycles, arising from the interaction between infection in primary producers and ecosystem nutrient recycling, as described below.

1a $$\begin{aligned} \frac{dS}{dt}=r\Bigg (1-\frac{S+I}{\min \{\frac{Kr}{r-\delta }, \frac{N_S(S+I)}{qS}\}}\bigg )S-\beta SI-\delta S, \hspace{100pt} \end{aligned}$$

1b $$\begin{aligned} \frac{dI}{dt}=\beta SI-\delta I-\nu I, \hspace{215pt} \end{aligned}$$

1c $$\begin{aligned} \frac{dN_S}{dt}=u(N_E)S-Q_S \beta SI-\delta N_S, \hspace{165pt} \end{aligned}$$

1d $$\begin{aligned} \frac{dN_I}{dt}=u(N_E)I+Q_S \beta SI-\delta N_I-\nu N_I, \hspace{145pt} \end{aligned}$$

1e $$\begin{aligned} \frac{dN_E}{dt}=-u(N_E)S-u(N_E)I+\delta (N_S+N_I)+\nu N_I, \hspace{100pt} \end{aligned}$$

where

2 $$\begin{aligned} Q_S=\frac{N_S}{S} \quad ,Q_I=\frac{N_I}{I} \quad \text {and} \quad u(N_E)=\frac{c N_E}{a+N_E}, \end{aligned}$$

*S* and *I* denote the densities of susceptible and infected hosts, measured in kg $C/m^2$, while $N_S$, $N_I$, and $N_E$ represent nutrients in susceptible hosts, infected hosts, and the environment, measured in g $N/m^2$, respectively. $Q_S$ and $Q_I$ correspond to the host stoichiometric quotas. The nutrient uptake function $u(N_E)$ follows a Holling type II functional response, as defined in 2, where *c* is the maximum N:C uptake rate and *a* is the half saturation constant of the N:C uptake. $\beta$ is the infection transmission rate, *q* is the minimum N:C ratio of the host, $\delta$ is the natural death rate of C, $\nu$ is the disease-induced death rate of C, and *r* is the maximum growth rate. The model assumes density-dependent pathogen transmission (Borer et al. 2021). It further assumes that the total nutrient concentration *N* equals the sum of nutrient concentrations in susceptible and infected autotrophs ($N_S$ and $N_I$, respectively) and in the environment ($N_E$), *i.e.*, $N = N_S + N_I + N_E$.

The minimum operator in the host growth term reflects Liebig’s law of the minimum, a central principle in ecological stoichiometry stating that growth is constrained by the most limiting resource (Sterner and Elser 2002). In this formulation, host growth switches between two regimes. When internal nutrient quotas are limiting, growth is constrained by nutrient availability (nutrient-limited regime). When nutrients are sufficiently abundant, growth is instead constrained by carbon availability and the logistic carrying capacity (carbon/light-limited regime). This regime switching introduces a nonlinear threshold structure into the model, as changes in environmental nutrient levels can shift the dominant limiting factor and thereby influence stability and disease dynamics.

Building on this stoichiometric foundation, we next introduce two targeted extensions that incorporate additional ecological realism into the framework. First, we modify model (1) by assuming nutrient-dependent uptake functions are parameterized differently depending on the health status of the host, $u_S(N_E)$ and $u_I(N_E)$. These follow Holling type II functional response, similar to above, where $c_S$, $c_I$ are maximum N:C uptake rates and $a_S$, $a_I$ are half saturation constants of the N:C uptake, for *S* and *I* hosts respectively.

Secondly, we incorporate a nutrient-driven disease transmission rate, $\beta (N_E)$. We assume that the nutrient concentration in the environment ($N_E$) exerts reciprocal effects on disease transmission through plant-soil interactions. When nutrients are limited, plants may produce more fine roots and/or spread them over larger areas to forage for scarce resources. This can increase overlap among neighboring plants, creating more contact points and shared soil pathways, which in turn will enhance transmission of pathogens transmitted through root-to-root contact. Specifically, we define the transmission rate $\beta (N_E)$ as a decreasing function of $N_E$,

3 $$\begin{aligned} \beta (N_E)=\frac{\beta _{\max }}{1+\kappa N_E} \end{aligned}$$

where $\beta _{\max }$ is the maximum transmission rate and $\kappa$ is the shape parameter. We parameterize $\beta _{\max }$, calibrating it so that our transmission function $\beta (N_E)$ is within the range of constant transmissions used in model (1) from Borer et al. (2022), see Figure 2.

**Fig. 1:**
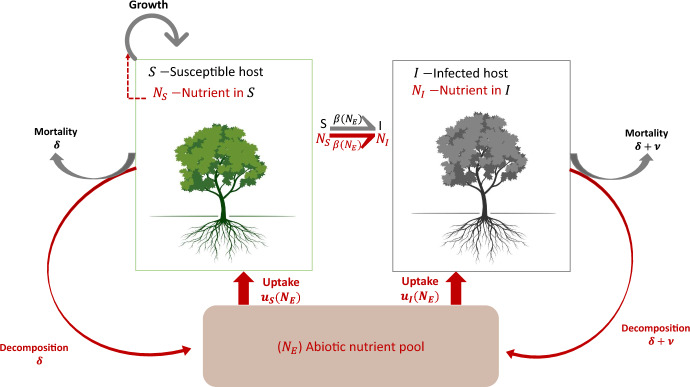
Simple visualizations of nutrient-driven transmission model (4) based on plant-soil interactions showing flows of C (grey arrows) and N (red arrows). The model tracks C and N among susceptible hosts (*S* and $N_S$) and infected hosts (*I* and $N_I$), as well as the abiotic nutrient levels ($N_E$). It includes nutrient-dependent rates for growth and transmission $\beta (N_E)$ (color figure online)

**Fig. 2:**
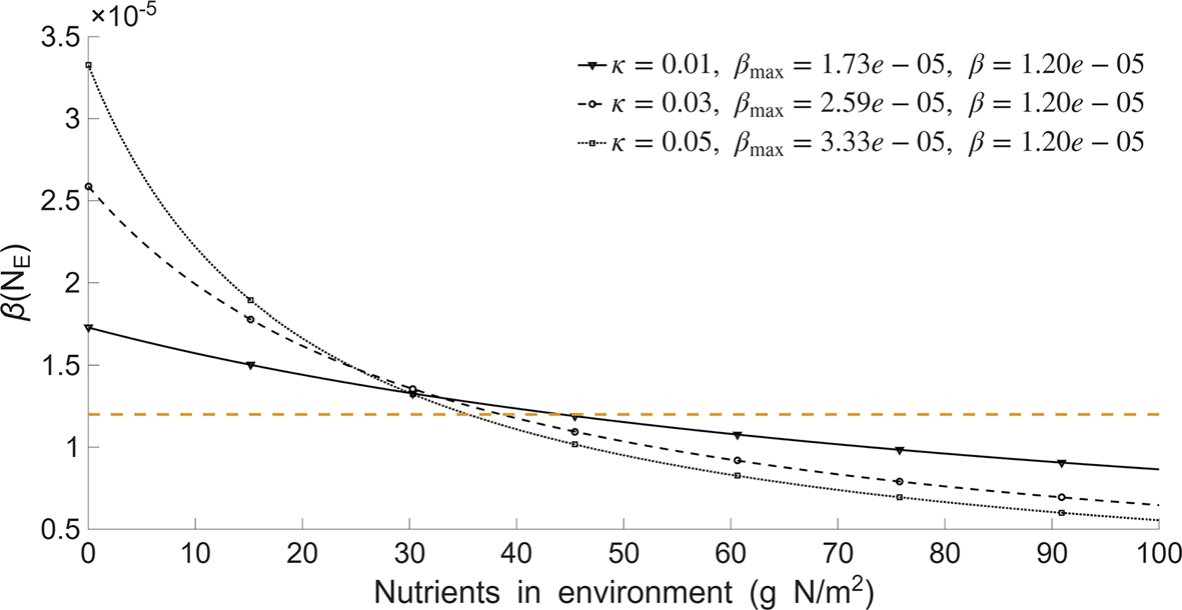
Nutrient-driven transmission rate, $\beta (N_E)$, as defined in equation (3), for varying values of $\kappa$ and $\beta _{\max }$ calibrated such that $\mathbb {E}[\beta (N_E)] = \beta = 1.2 \times 10^{-5}$ (from the model (1); Borer et al. (2022)) for intermediate N values (color figure online)

Incorporating these modifications into model (1) leads to our nutrient-driven transmission rate model (Fig. 1), given by:

4a $$\begin{aligned} \frac{dS}{dt}=r\Bigg (1-\frac{S+I}{\min \{\frac{Kr}{r-\delta }, \frac{N_S(S+I)}{qS}\}}\bigg )S-\beta (N_E) SI-\delta S,\hspace{100pt} \end{aligned}$$

4b $$\begin{aligned} \frac{dI}{dt}=\beta (N_E) SI-\delta I-\nu I, \hspace{215pt} \end{aligned}$$

4c $$\begin{aligned} \frac{dN_S}{dt}=u_S(N_E)S-Q_S \beta (N_E) SI-\delta N_S, \hspace{160pt} \end{aligned}$$

4d $$\begin{aligned} \frac{dN_I}{dt}=u_I(N_E)I+Q_S \beta (N_E) SI-\delta N_I-\nu N_I, \hspace{140pt} \end{aligned}$$

4e $$\begin{aligned} \frac{dN_E}{dt}=-u_S(N_E)S-u_I(N_E)I+\delta (N_S+N_I)+\nu N_I, \hspace{110pt} \end{aligned}$$

where

5 $$\begin{aligned} \beta (N_E)=\frac{\beta _{\max }}{1+\kappa N_E} , \quad u_S(N_E)=\frac{c_S N_E}{a_S+N_E}, \quad \text {and} \quad u_I(N_E)=\frac{c_I N_E}{a_I+N_E}. \end{aligned}$$

## Model Analysis

We begin by analyzing our nutrient-driven transmission model (4) to prove that its solutions remain positive and bounded within biologically realistic ranges, as established by the following theorems.

### Theorem 1

(Positivity) Solution to the system (4) with initial conditions in the set

$$\begin{aligned}\Omega _1=\bigg \{(S,I,N_S,N_I,N_E):0\le S,0\le I, 0\le N_S, 0\le N_I, 0\le N_E \bigg \}\end{aligned}$$

will remain there for all forward time.

### Proof

Let $\mathbb {D}(t)=(S(t), I(t),N_S(t),N_I(t),N_E(t))$ be a solution of system (4) with $\mathbb {D}(0) \in \Omega _1$. Assume that $\mathbb {D}(t_1)$ touches or crosses a boundary of $\Omega _1$ for the first time for some $t_1 >0$. We prove this in the following cases.

- **Case 1:** If $S(t_1)=0$ for $t_1 >0$, then $\frac{dS}{dt}|_{t=t_1}=0$, and hence $S(t) = 0$ for all $t>t_1>0$ or we can say $S(t)\ge 0$ for all $t>0$.
- **Case 2:** If $I(t_1)=0$ for $t_1 >0$, then $\frac{dI}{dt}|_{t=t_1}=0$, and hence $I(t)=0$ for all $t>t_1>0$ or we can say $I(t)\ge 0$ for all $t>0$.
- **Case 3:** If $N_S(t_1)=0$ for $t_1 >0$. Since $S(t_1) \ge 0$, $N_E(t_1) \ge 0$ and $Q_S(t_1)=\frac{N_S(t_1))}{S(t_1))}=0$, then $\frac{dN_S}{dt}|_{t=t_1} \ge 0$, and hence $N_S(t)\ge 0$ for all $t>t_1>0$ or we can say $N_S(t)\ge 0$ for all $t>0$.
- **Case 4:** If $$N_I(t_1)=0 $$ for $$t_1 >0$$. Since $$S(t_1) \ge 0$$, $$I(t_1) \ge 0$$, $$N_S(t_1) \ge 0$$, $$N_E(t_1) \ge 0$$ and $$Q_S(t_1)=\frac{N_S(t_1))}{S(t_1))}\ge 0$$, then $$\frac{dN_I}{dt}|_{t=t_1} \ge 0$$, and hence $$N_I(t)\ge 0$$ for all $$t>t_1>0$$ or we can say $$N_I(t)\ge 0$$ for all $$t>0$$.
- **Case 5:** If $$N_E(t_1)=0 $$ for $$t_1 >0$$, $$u_S(N_E)(t_1)=0$$, $$u_I(N_E)(t_1)=0$$. Since $$N_I(t_1) \ge 0$$, $$N_S(t_1) \ge 0$$, then $$\frac{dN_E}{dt}|_{t=t_1} \ge 0$$, and hence $$N_E(t)\ge 0$$ for all $$t>t_1>0$$ or we can say $$N_E(t)\ge 0$$ for all $$t>0$$.

Therefore, from the above discussion, we conclude that no trajectory can leave the positively invariant set $\Omega _1 \subset \mathbb {R}_+^5$; that is, any solution that starts in $\Omega _1$ remains in $\Omega _1$ for all $t \ge 0$. $\square$

### Theorem 2

(Invariance of Solutions) Solution to the system (4) with initial conditions in the positive and bounded set

$$\begin{aligned} \Omega \!\!=\!\!\bigg \{(S,I,N_S,N_I,N_E): 0\!\le \! S, I, N_S, N_I, N_E, S\!+\!I \!\le \! \overline{K}, N_S\!+\!N_I\!+\!N_E\!=\!N\!<\!\infty \bigg \} \end{aligned}$$

where $\overline{K}=\min \bigg \{\frac{Kr}{r-\delta }, \frac{Q_S(S+I)}{q}\bigg \}$, will remain there for all forward time.

### Proof

Let $\mathbb {D}(t)=(S(t), I(t),N_S(t),N_I(t),N_E(t))$ be a solution of system (4) with $\mathbb {D}(0) \in \Omega$. From Theorem 1, we proved that the solution $\mathbb {D}(t)$ remains nonnegative for all $t\ge 0$. Now, we prove the boundedness of the solution so that any solution starting in $\Omega$ remains in $\Omega$ for all $t\ge 0$. We prove this in the following cases by contradiction.

- **Case 1:** Suppose that solutions leave $\Omega$ for the first time at $t_1 > 0$ through the boundary $N_S+N_I+N_E=N$. Then for $t_1 > 0$ we have
  6 $$\begin{aligned} N_S+N_I+N_E> N, \hspace{5pt} \text {for}\hspace{5pt} t_1>0. \end{aligned}$$
  Since for $t\in [0,t_1)$, $N_S+N_I+N_E=N$, implies that
  7 $$\begin{aligned} N_S'(t_1)+N_I'(t_1)+N_E'(t_1) > 0. \end{aligned}$$
  Using 4c, 4d, 4e, we obtained
  $$\begin{aligned} N_S'(t_1)+N_I'(t_1)+N_E'(t_1)=0 \end{aligned}$$
  This contradicts (7) and completes the proof.
- **Case 2:** Suppose that solutions crosses $\Omega$ for the first time at $t_1 > 0$ through the boundary $S+I = \min \bigg \{\frac{Kr}{r-\delta }, \frac{Q_S(S+I)}{q}\bigg \}$. Then at $t_1$ we have
  8 $$\begin{aligned} S+I = \min \bigg \{\frac{Kr}{r-\delta }, \frac{Q_S(S+I)}{q}\bigg \} \end{aligned}$$
  and
  9 $$\begin{aligned} S'(t_1)+I'(t_1) \ge 0. \end{aligned}$$
  However, at $t=t_1$ we obtain the following:
  $$\begin{aligned}&S'(t_1)+I'(t_1)\\&=r\Bigg (1-\frac{S+I}{\min \{\frac{Kr}{r-\delta }, \frac{N_S(S+I)}{qS}\}}\bigg )S-\delta (S+I)-\nu I\\&=r\Bigg (1-\frac{S+I}{\min \{\frac{Kr}{r-\delta }, \frac{Q(S+I)}{q}\}}\bigg )S-\delta (S+I)-\nu I\\&=-\delta (S+I)-\nu I, \text {by using}\hspace{5pt} \hbox {(8)} \\&< 0 \end{aligned}$$
  This contradicts (9) and completes the proof.

$$\square $$

### Disease-free Equilibria

Our model incorporates threshold minimum functions to capture the effects of nutrient availability on host growth rates, following Justus von Liebig’s law of the minimum (Sterner and Elser 2002). The general form of the disease-free equilibria (DFEs) of model (4) is given by: 810

$$\begin{aligned}&(\overline{S}, \overline{I}, \overline{N_S}, \overline{N_I}, \overline{N_E}) \\ \quad&\!\!=\!\!\Bigg (\min \left\{ K,\; \frac{c_S (r-\delta )\,\overline{N_E}}{r q \delta \,(a_S+\overline{N_E})} \right\} , 0,\min \left\{ K,\; \frac{c_S (r-\delta )\,\overline{N_E}}{r q \delta \,(a_S+\overline{N_E})} \right\} \frac{c_S \overline{N_E}}{\delta (a_S+\overline{N_E})}, 0, \overline{N_E} \Bigg ) \end{aligned}$$

We assume $r>\delta$ so $\overline{S}>0$. Here $\overline{N_E}$ will depend on *K*, as well as the total amount of nutrients in the system, which is given in the initial conditions $N=N_S(0)+N_I(0)+N_E(0)$. The form for $\overline{N_E}$ depends on the cases discussed below. Considering the threshold minimum functions, model (4) admits distinct DFEs with the cases:

- **Case 1:** If $\overline{S}=K$ , the model (4) admits the DFE
  10 $$\begin{aligned} (\overline{S}, \overline{I}, \overline{N_S}, \overline{N_I}, \overline{N_E})= \Bigg (K, 0, \frac{c_S K N_E^*}{\delta (a_S+N_E^*)},0, N_E^* \Bigg ). \end{aligned}$$
  Using the assumption that the total nutrient concentration *N* equals the sum of nutrient concentrations in both autotrophs ($N_S$ and $N_I$) and the environment($N_E$), we have
  $$\begin{aligned}N_E^*=N-\overline{N_S}-\overline{N_I}\end{aligned}$$
  Substituting this into (10) gives two possible DFEs:
  $$\begin{aligned}E_i=\left( K,0,\omega _i,0,N-\omega _i\right) ,\quad i=1,2\end{aligned}$$
  which exist under the conditions
  $$\begin{aligned}\phi ^2 \ge 4 \delta c_s K N .\end{aligned}$$
  where
  $$\begin{aligned}\phi =c_s K+\delta (N+a_s)>0, \qquad \omega _i=\frac{\phi \pm \sqrt{\phi ^2-4 \delta c_sK N}}{2\delta }>0. \end{aligned}$$
  Therefore, Two possible Disease-free Equilibria (DFEs) of the system (4) are
  $$\begin{aligned} \left( K, 0, \tfrac{\phi + \sqrt{\phi ^2-4 \delta c_s K N}}{2\delta },0, N-\tfrac{\phi + \sqrt{\phi ^2-4 \delta c_s K N}}{2\delta }\right) \end{aligned}$$
  and
  $$\begin{aligned} \left( K, 0,\tfrac{\phi - \sqrt{\phi ^2-4 \delta c_s K N}}{2\delta },0, N-\tfrac{\phi - \sqrt{\phi ^2-4 \delta c_s K N}}{2\delta }\right) \end{aligned}$$
  where,
  $$\begin{aligned} \phi =c_s K+\delta (N+a_s)>0 \quad \text {with}\quad \phi ^2 \ge 4 \delta c_s K N\end{aligned}$$
  However, the DFE,
  $$\begin{aligned} \left( \overline{S}, 0, \overline{N_S}, 0, \overline{N_E}\right) = \left( K, 0, \tfrac{\boldsymbol{\phi } + \sqrt{\phi ^2-4 \delta c_sK N}}{2\delta },0, N-\tfrac{\phi + \sqrt{\phi ^2-4 \delta c_sK N}}{2\delta }\right) \end{aligned}$$
  is not biologically feasible because $\overline{N_{E}}<0$ (Appendix. Appendix C).
- **Case 2:** If $\overline{S}=\frac{c_S (r-\delta ) \overline{N_E}}{rq\delta (a_S+\overline{N_E})}$ with $r>\delta$, the Model (4) admits the DFE
  11 $$\begin{aligned} (\overline{S}, \overline{I}, \overline{N_S}, \overline{N_I}, \overline{N_E})==\left( \frac{c_S (r-\delta ) N_E^{**} }{rq\delta (a_S+N_E^{**} )},0,\frac{c_S N_E^{**} }{\delta (a_S+ N_E^{**}) }\overline{S}, 0,N_E^{**} \right) \end{aligned}$$
  Using $$ N_E^{**} =N- \overline{N_S}$$ in (11), we obtain
  $$\begin{aligned}\left( \overline{S}, 0, \overline{N_S}, 0, \overline{N_E}\right) =\left( \frac{(r-\delta )(N- N_E^{**})}{rq},0,N- N_E^{**}, 0, N_E^{**} \right) \end{aligned}$$
  where
  $$\begin{aligned} N_E^{**}=\frac{\delta rqa_s}{c_s (r-\delta )-\delta rq}\qquad \text {and} \qquad c_s (r-\delta ) \ge \delta rq\end{aligned}$$

Therefore, The Disease-free Equilibria (DFEs) of the system (4) are

12 $$\begin{aligned} E_1=\left( \overline{S}, 0, \overline{N_S}, 0, \overline{N_E}\right) = \left( K, 0,\tfrac{\phi - \sqrt{\phi ^2-4 \delta c_sK N}}{2\delta },0, N-\tfrac{\phi - \sqrt{\phi ^2-4 \delta c_sK N}}{2\delta }\right) \end{aligned}$$

$$\text {for} \quad K \le \frac{c_S (r-\delta ) \overline{N_E}}{rq\delta (a_S+\overline{N_E})},$$ where, $$\phi =c_s K+\delta (N+a_s)>0 \quad \text {with}\quad \phi ^2 \ge 4 \delta c_s K N $$

and

13 $$\begin{aligned} E_2=\left( \frac{(r-\delta )(N- \frac{\delta rqa_s}{c_s (r-\delta )-\delta rq})}{rq},0,N- \frac{\delta rqa_s}{c_s (r-\delta )-\delta rq}, 0, \frac{\delta rqa_s}{c_s (r-\delta )-\delta rq} \right) \end{aligned}$$

$$\text {for} \quad K > \frac{c_S (r-\delta ) \overline{N_E}}{rq\delta (a_S+\overline{N_E})} $$, where, $$c_s (r-\delta ) \ge \delta rq \quad \text {with}\quad r>\delta $$

#### Theorem 3

The Disease-free Equilibria (DFEs) $E_1$ and $E_2$, whenever they exist, (see Subsection 3.1) of the system (4), are locally asymptotically stable if $\mathcal {R}_0<1$, and unstable if $\mathcal {R}_0>1$, where $\mathcal {R}_0$ for a DFE $\left( \overline{S}, 0,\overline{N_S},0,\overline{N_E}\right)$ is defined by

14 $$\begin{aligned} \mathcal {R}_0=\left[ \frac{\beta (\overline{N_E})}{\delta +\nu } \overline{S}\right] \end{aligned}$$

In particular,

$$\begin{aligned} \mathcal {R}_0(E_1) = \left[ \frac{\beta (\overline{N_E})}{\delta +\nu }\,\overline{S}\right] \quad \text {for} \quad K \le \frac{c_S (r-\delta ) \overline{N_E}}{rq\delta (a_S+\overline{N_E})}, \end{aligned}$$

and

$$\begin{aligned} \mathcal {R}_0(E_2)=\left[ \frac{\beta (\overline{N_E})}{\delta +\nu } \overline{S}\right] \quad \text {for} \quad K > \frac{c_S (r-\delta ) \overline{N_E}}{rq\delta (a_S+\overline{N_E})} \end{aligned}$$

where the corresponding values of $\overline{N_E}$ and $\overline{S}$ are given explicitly in Eq. (12) and Eq. (13) respectively .

#### Proof

The basic reproduction number for the infected host was computed using the next-generation matrix method.Model (4) has a single infected compartment:

$$\begin{aligned}\frac{dI}{dt}=\beta (N_E) SI-\delta I-\nu I, \text {where}\quad \beta (N_E)=\frac{\beta _{max}}{1+\kappa N_E}\end{aligned}$$

Consider this equation written in the form:

$$\begin{aligned}\frac{dI}{dt}=\left[ \frac{\beta _{max}}{1+\kappa N_E} SI\right] -\left[ (\delta +\nu )I\right] \end{aligned}$$

Now, define

$$\begin{aligned}F=\frac{\partial }{\partial I}\left[ \frac{\beta _{max}}{1+\kappa N_E} SI\right] =\left[ \frac{\beta _{max}}{1+\kappa N_E} S\right] \text {and} \quad V=\frac{\partial }{\partial I}\left[ (\delta +\nu )I\right] =\left[ \delta +\nu \right] \end{aligned}$$

by evaluating *F* and *V* at DFE: $\left( \overline{S}, 0,\overline{N_S},0,\overline{N_E}\right) ,$

$$\begin{aligned}F=\left[ \frac{\beta _{max}}{1+\kappa \overline{N_E}} \overline{S}\right] \quad \text {and} \quad V=\left[ \delta +\nu \right] \end{aligned}$$

Note that $J=F-V$ is the Jacobian matrix of the ODES system (4) evaluated at the DFE.We can define next generation matrix as

$$\begin{aligned}FV^{-1}=\frac{1}{\delta +\nu }\left[ \frac{\beta _{max}}{1+\kappa \overline{N_E}} \overline{S}\right] =\left[ \frac{\beta (\overline{N_E})}{\delta +\nu } \overline{S}\right] \quad \text {where} \quad \beta (\overline{N_E})=\frac{\beta _{max}}{1+\kappa \overline{N_E}}\end{aligned}$$

Hence, Basic Reproduction Number

15 $$\begin{aligned} \mathcal {R}_0=\rho (FV^{-1})=\left[ \frac{\beta (\overline{N_E})}{\delta +\nu } \overline{S}\right] \end{aligned}$$

Therefore, applying Theorem 2 of Driessche and Watmough (2002), the Disease-free Equilibria (DFEs) $E_1$ and $E_2$ of the system (4) are locally asymptotically stable if $\mathcal {R}_0<1$, and unstable if $\mathcal {R}_0>1$. $\square$

The expression for $\mathcal {R}_0$ 15 reveals that disease invasion depends jointly on transmission efficiency and nutrient-driven host abundance. In particular, nutrient availability influences $\mathcal {R}_0$ indirectly through the equilibrium susceptible population $\overline{S}$ and directly through the transmission function $\beta (\overline{N_E})$. Thus, nutrient enrichment can either facilitate or suppress disease spread depending on parameter regimes.

It is worth noting that the stability results established here are local, as they are based on linearization around the equilibrium points. Due to the nonlinear structure of the model, particularly the nutrient-dependent transmission term and the threshold minimum functions, a comprehensive global analysis is beyond the scope of the present study. Nevertheless, the local results provide valuable insight into the behavior of solutions in neighborhoods of the equilibria, and further investigation of the global dynamics remains an interesting direction for future research.

### Endemic Solutions

**Fig. 3:**
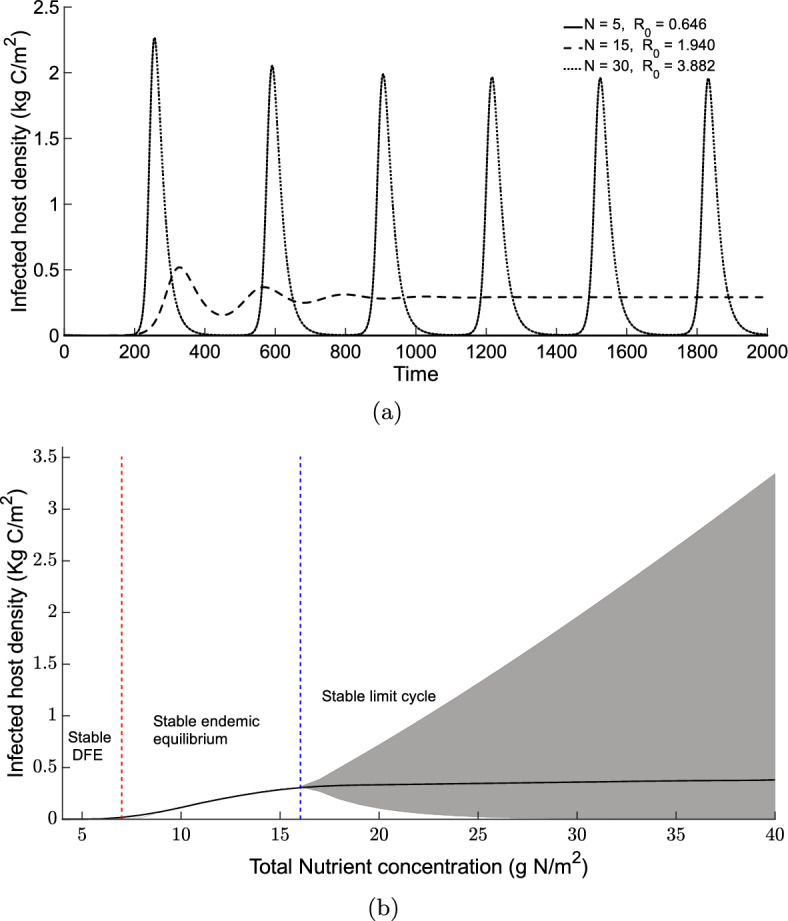
Numerical simulation (a) and bifurcation diagram (b) of model (4), showing the infected host density (*I*) for varying total nutrient concentrations (*N*), using the parameter values listed in Table 1. The bifurcation diagram illustrates the long-term dynamics ($t > 20{,}000$ years), where curves show steady-state dynamics, shaded regions depict the maximum and minimum values of limit cycles, and curves inside shaded regions show average values of the limit cycles (color figure online)

The model (4) admits stable solutions, both equilibrium and limit cycles, depending on parameter values. However, due to the model complexity, obtaining an explicit analytical expression for these is challenging. Therefore, we investigate the existence and local stability of the endemic solutions numerically (Figure 3).

Figure 3a illustrates the numerical simulations of the infected host density (*I*) under three different total nutrient concentrations, $N = 5~\text {g N/m}^2$, $15~\text {g N/m}^2$, and $30~\text {g N/m}^2$, while Figure 3b shows the corresponding bifurcation structure of the infected host density (*I*) as the total nutrient concentration (*N*) varies. For $N = 5~\text {g N/m}^2$, the infected population becomes extinct. Since the basic reproduction number satisfies $\mathcal {R}_0 < 1$, the disease-free equilibrium (DFE) is locally asymptotically stable. This stable disease-free regime is also reflected in the bifurcation diagram for nutrient levels where $\mathcal {R}_0 < 1$. As the nutrient level increases beyond the endemic emergence threshold, which is defined as the nutrient concentration at which the endemic equilibrium first appears ($I>0$) and is indicated by the red dashed line in Figure 3b, the disease-free equilibrium loses stability and a stable endemic equilibrium emerges. For example, at $N = 15~\text {g N/m}^2$, where $\mathcal {R}_0 = 1.94$, the disease persists and the endemic equilibrium is locally asymptotically stable. As nutrient levels increase further beyond the blue dashed line in Figure 3b, the endemic equilibrium loses stability and sustained oscillations emerge. The bifurcation diagram indicates the presence of a stable limit cycle in this regime. For example, at $N = 30~\text {g N/m}^2$ ($\mathcal {R}_0 = 3.882$), the disease persists and exhibits periodic outbreaks.

## Numerical Analysis

This section describes the results of numerical experiments and numerical bifurcation analyses of the nutrient-driven transmission model (4). The initial conditions and several parameter values were adopted from models previously developed by Borer et al. (2022, 2021). Initial conditions for all simulations presented were $S(0) = 1$ kg $C/m^2$, $I(0) = 0.001$ kg $C/m^2$, $N_S(0) = q \times S(0)$ g $N/m^2$, $N_I(0) = q \times I(0)$ g $N/m^2$, and $N_E(0) = (N - N_S(0) - N_I(0))$ g $N/m^2$. In our model, we assume different nutrient uptake functions, $u_S(N_E)$ and $u_I(N_E)$, for susceptible and infected hosts, respectively, as defined in (5). The parameter $\beta _{\max }$ is calibrated such that $\mathbb {E}[\beta (N_E)] = \beta$, where $\beta$ denotes the transmission rate in model (1).

In addition to illustrating transient dynamics, the numerical analysis is used to explore parameter regimes, regime transitions, and sensitivity to key model parameters. In particular, we examine how variations in total nutrient concentration, transmission intensity, and light-dependent carrying capacity influence disease persistence and oscillatory behavior. These simulations complement the local analytical results by characterizing bifurcation structures and identifying dominant drivers of $\mathcal {R}_0$.

**Fig. 4:**
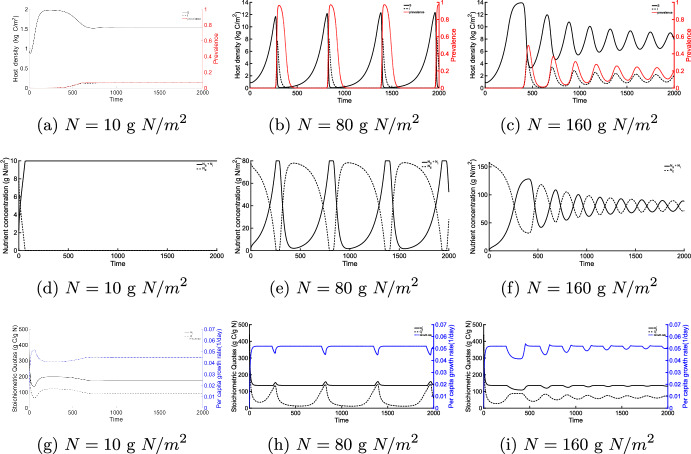
Numerical simulation of model (4) for varying values of *N* representing (left) low-nutrient condition ($N=10$ g $N/m^2$), (middle) medium-nutrient condition ($N=80$ g $N/m^2$), and (right) high-nutrient condition ($N=160$ g $N/m^2$), showing host density and prevalence (a,b,c); nutrient concentration in the host and environment (d,e,f); and growth rate and stoichiometric quota (g,h,i). Simulations use the parameter values listed in Table 1, under C-dependent carrying capacity $K=14$ kg $C/m^2$ (color figure online)

**Fig. 5:**
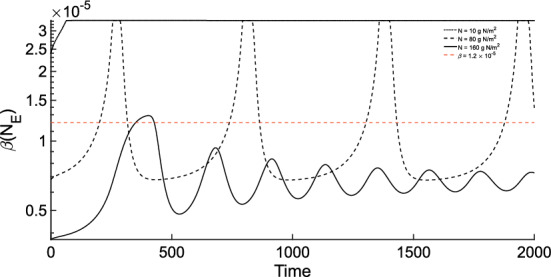
Numerical simulation of model (4), showing $\beta (N_E)$. Simulations use the parameter values listed in Table 1, under C-dependent carrying capacity $K=14$ kg $C/m^2$ with varying values *N*. These simulations correspond to those shown in Fig. 4 (color figure online)

Here, the model parameters are based on a deciduous forest ecosystem, and numerical simulations were conducted to examine the dynamic consequences and magnitude of changes arising from disease–nutrient interactions, see Table 1. Most parameter values are drawn from previously published studies on nutrient-host systems in deciduous forest ecosystems (Borer et al. 2021, 2022). Where direct empirical estimates were unavailable, parameter values were selected to remain biologically plausible and consistent with the scaling of the model. To assess robustness, we further performed sensitivity and bifurcation analyses over biologically meaningful parameter ranges. Because trees are long-lived hosts, simulations of transient temporal dynamics were run over a 2000-year period. In Figure 4, we show how host population density, host stoichiometry, growth rate, infection prevalence, and the distribution of nutrients between autotrophs and the environment respond under three different nutrient conditions: low ($N = 10$ g $N/m^2$), medium ($N = 80$ g $N/m^2$), and high ($N = 160$ g $N/m^2$). At a low nutrient level ($N = 10$ g $N/m^2$), the model dynamics approach a stable equilibrium with low infection prevalence and nutrient levels peak in autotrophs, while environmental nutrients reach a minimum, and host stoichiometry and growth rate stabilize (Figs. 4a, 4d, 4g). Higher values of *N* yield oscillatory dynamics, with stable limit cycles. Infection prevalence also oscillates with high amplitude.(Figs. 4b, 4e, 4h). For even higher values of *N*, the model dynamics exhibit oscillations that gradually dampen (Figs. 4c, 4f, 4i). These dynamics arise from the nutrient-driven disease transmission rate, $\beta (N_E)$. At low nutrient conditions, the environmental nutrient concentration stabilizes at a minimal level(Fig. 4d), resulting in a maximal transmission rate via $\beta (N_E)$. In contrast, at higher nutrient conditions, environmental nutrient concentrations exhibit large-amplitude oscillations, causing $\beta (N_E)$ to fluctuate accordingly (Fig. 5).

**Table 1:** Nutrient-driven transmission model (4) parameter table

Parameter	Description	Baseline	Source
*r*	maximum growth rate	0.0754 /year	Borer et al. (2021)
*K*	C-dependent carrying capacity	14 kg $C/m^2$	Seabloom et al. (2023)^*^
*q*	minimum N: C ratio of host	1/439 g N/g C	Borer et al. (2021)
$\delta$	C natural death rate	0.0412 /year	Borer et al. (2021)
$\nu$	C disease-induced death rate	0.01 /year	Borer et al. (2022)
$\beta _{\max }$	maximum transmission rate	$3.33\times 10^{-5}m^2$/g C/year	estimated
$c_S$	maximum N:C uptake rate of Susceptible host	$3.8\times 10^{-4}$ g N/g C/year	Borer et al. (2021)
$$a_S $$	N:C uptake half saturation constant of Susceptible host	0.009 g $$N/m^2$$	Borer et al. (2022)
$c_I$	maximum N:C uptake rate of Infected host	$1.001\times c_S$ g N/g C/year	Borer et al. (2021)
$$a_I $$	N:C uptake half saturation constant of Infected host	0.009 g $$N/m^2$$	Borer et al. (2022)
$\kappa$	shape parameter/decay rate	0.05 $m^2$/g N	assumed

*The baseline value of parameter *K* is 14 kg $C/m^2$, based on the range of C-dependent carrying capacity (*K* = 1–18 kg $C/m^2$) reported in Seabloom et al. (2023)

### $\mathcal {R}_0$ Sensitivity analysis

To quantify parameter influence on disease invasion, we performed a Latin Hypercube Sampling (LHS) combined with Partial Rank Correlation Coefficient (PRCC) analysis, focusing on the basic reproduction number $\mathcal {R}_0$. This allows identification of the most influential parameters that govern disease emergence in biologically relevant ranges. All 11 parameters in Table 1 and the total nutrient concentration *N* used in the initial conditions were treated as uncertain, and the LHS was performed with 10,000 simulations. Each parameter was sampled from a uniform distribution ranging from half to twice its baseline value (Table 1), except for *K* and *N*, which were assigned broader biologically plausible ranges.

**Fig. 6:**
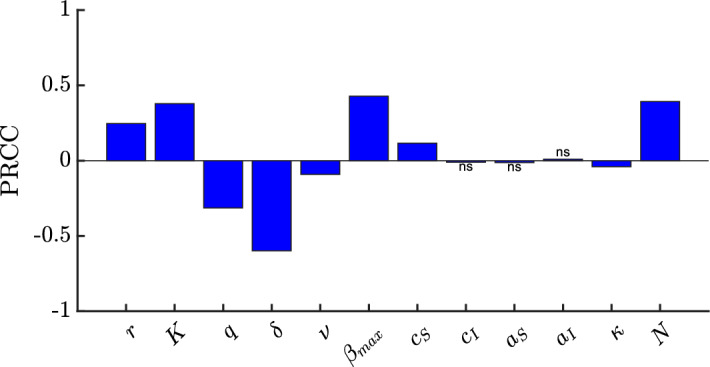
PRCC values for the basic reproduction number $\mathcal {R}_0$, sampling parameters in ranges from half to twice their baseline values listed in Table 1, using LHS sampling. Bars labeled “ns” denote non-significant effects ($p>0.05$). PRCC values range from -1 to 1, where negative values indicate an inverse relationship between the parameter and the output measure, and positive values suggest a direct positive impact on the output (color figure online)

Since the two disease-free equilibria $E_1$ and $E_2$ of system (4) cannot coexist for the same parameter set, the basic reproduction number $\mathcal {R}_0$ was computed at the disease-free equilibrium that exists under the corresponding parameter regime. The PRCC is an appropriate statistical measure when parameters are monotonically related to output measures (Marino et al. 2008). Therefore, we examined the relationship between each parameter and the output measure individually (Appendix Appendix B). When monotonic trends were observed, PRCC values were computed as sensitivity indices (Fig. 6).

Following Marino et al. (2008), a z-test was performed on the transformed PRCC values to evaluate statistical significance. Parameters with larger absolute PRCC values exert a stronger influence on the basic reproduction number $\mathcal {R}_0$. The PRCC results indicate that the parameters *r*, *K*, *q*, $\delta$, $\nu$, $\beta _{\max }$, $c_S$, $\kappa$, and *N* significantly influence the basic reproduction number $\mathcal {R}_0$. Negative PRCC values indicate an inverse relationship between the parameter and the basic reproduction number, whereas positive values indicate a direct relationship. For example, a negative PRCC value implies that increasing the parameter decreases the basic reproduction number, while a positive value implies that increasing the parameter increases the basic reproduction number. These results provide deeper insight into the nutrient disease feedback mechanisms within the model.

### Bifurcation Diagrams

**Fig. 7:**
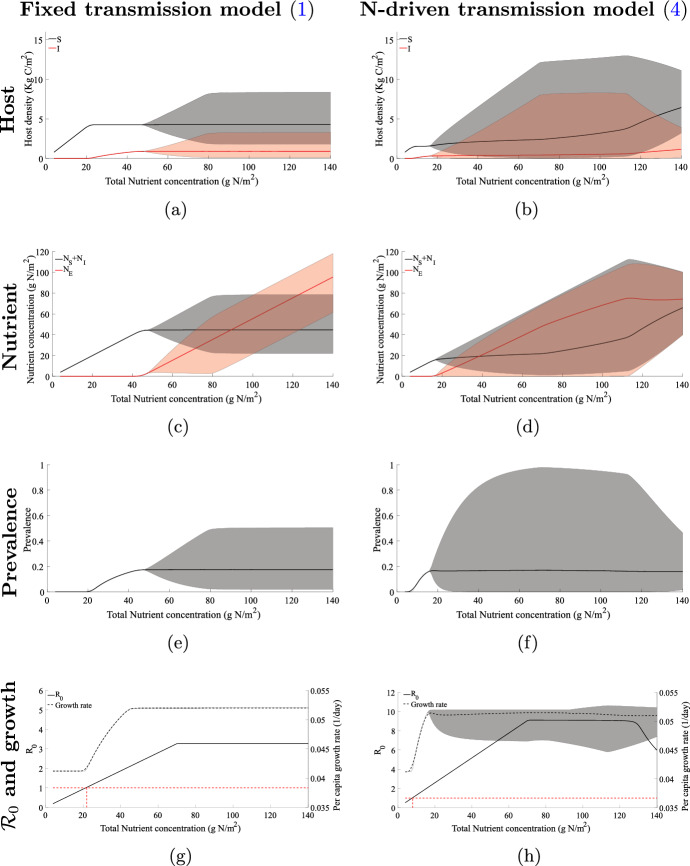
Bifurcation diagrams of model (1) (left) and model (4) (right), showing host density (a,b), nutrient concentration in the host (c,d), prevalence (e,f), and $\mathcal {R}_0$ and growth rate (g,h) for $K = 14 \text {kg C/m}^{2}$. Other parameter values are listed in Table 1, and bifurcations are shown for varying total nutrient concentrations (*N*). These diagrams illustrate the long-term dynamics ($t > 20{,}000$ years), where curves show steady-state dynamics, shaded regions depict the maximum and minimum values of limit cycles, and curves inside shaded regions show average values of the limit cycles (color figure online)

Bifurcation diagrams were constructed with respect to total nutrient input *N*, maximum transmission rate $\beta _{\max }$, and the light-dependent carrying capacity *K* to investigate regime shifts between disease extinction, endemic equilibria, and oscillatory dynamics. We conducted numerical bifurcation analyses to examine system dynamics across a range of nutrient levels by varying the total nutrient concentration (*N*). We compared the constant transmission model (1) with our nutrient-driven transmission model (4) under a carbon-dependent carrying capacity of $K = 14$ kg $C/m^2$. This comparison highlights how variation in the nutrient pool (*N*) influences population dynamics in both models (Fig. 7). In model (1), infection goes extinct for $N \le 20$ g $N/m^2$ with $\mathcal {R}_0<1$. A Hopf bifurcation arises near $N= 50$ g $N/m^2$, producing limit cycles that expand in amplitude for $50 \le N \le 80$ and then remain stable under nutrient-rich conditions. $\mathcal {R}_0$ rises with increasing nutrient concentration and levels off at high nutrient supply. The per capita host growth rate remains constant when $\mathcal {R}_0<1$, then increases with nutrient enrichment and plateaus at high *N* values. (Fig. 7: First column).

The bifurcation structures of model (1) and model (4) differ noticeably. In model (4), infection goes extinct under very low nutrient conditions ($N < 10$ g $N/m^2$) when $\mathcal {R}_0 < 1$. A Hopf bifurcation arises earlier ($N \le 20$ g $N/m^2$) than in model (1). As *N* increases, host infection and prevalence rise until a Hopf bifurcation generates limit cycles, which initially expand in amplitude and then weaken (Fig. 7, second column). In contrast, while model (1) also undergoes a Hopf bifurcation, the resulting limit cycles remain stable and persist indefinitely. In model (4), $\mathcal {R}_0$ increases with nutrient concentration, reaches a plateau under nutrient-rich conditions, and then declines at higher nutrient levels (Fig. 7h), while in model (1), $\mathcal {R}_0$ levels off around 3 under nutrient-rich conditions (Fig. 7g). We see similar patterns in the growth rate dynamics of both models, with constant per capita growth when $\mathcal {R}_0<1$, then increasing and saturating growth rates as *N* increases; however, the oscillations in the growth rates are large in amplitude for model (4) (Figs. 7g, 7h). The observed patterns of extinction, amplification, and decline emphasize that model (4), incorporating nutrient-driven transmission, is more sensitive to nutrient variation than model (1), which employs a constant transmission rate. These differences indicate that incorporating nutrient-dependent transmission fundamentally alters how enrichment shapes disease dynamics, introducing feedbacks that can both amplify and suppress infection across the nutrient gradient.

#### Nutrient–Disease Feedbacks

**Fig. 8:**
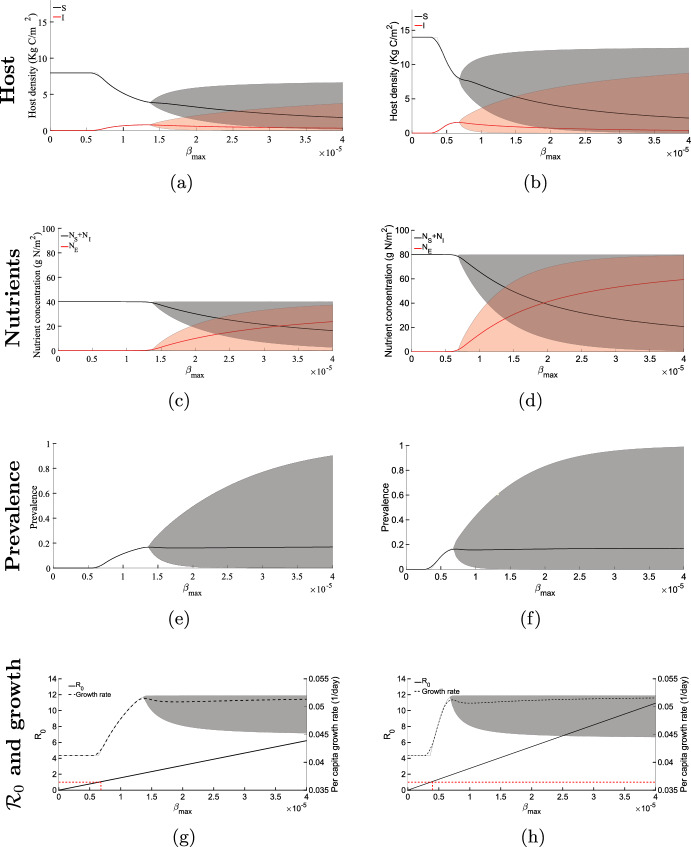
Bifurcation diagram of model (4) for varying values of $\beta _{\max }$ under two ecosystem nutrient levels: (left) $N=40$ kg $N/m^2$(Low nutrient condition), and (right) $N=80$ kg $N/m^2$ (High nutrient condition), showing: host density (a,b), nutrient concentration in the host (c,d), prevalence (e,f), and basic reproduction number $\mathcal {R}_0$ and growth rate (g,h). Parameter values are listed in Table 1. These bifurcation diagrams illustrate the long-term dynamics ($t > 20{,}000$ years), where curves show steady-state dynamics, shaded regions depict the maximum and minimum values of limit cycles, and curves inside shaded regions show average values of the limit cycles (color figure online)

Nutrient-disease feedbacks capture the bidirectional interactions in which nutrient availability influences disease dynamics, while disease, in turn, alters nutrient cycling and host growth. These effects can significantly reshape the dynamics of hosts, pathogens, and nutrients. We conducted numerical bifurcation analyses to examine system dynamics across a range of maximum transmission rates ($\beta _{\text {max}}$) under low and high nutrient levels (Fig. 8). Under low nutrient conditions ($N = 40$ g $N/m^2$), the threshold value of $\beta _{\text {max}}$ required for infection persistence was higher than under nutrient-rich conditions ($N = 80$ g $N/m^2$) (Fig. 8). This threshold corresponds to the bifurcation at which the disease-free and endemic equilibria exchange stability, coinciding with the basic reproduction number crossing 1. As $\beta _{\text {max}}$ increases, host infection and prevalence rise until a Hopf bifurcation generates limit cycles, which arise earlier under high nutrient conditions than under low nutrient conditions (Figs. 8a, 8b, 8e, 8f). Under high nutrient availability, the transition from stable nutrient dynamics to unstable cycles occurs at a smaller value of $\beta _{\text {max}}$ than under low nutrient conditions. Moreover, across the full range of $\beta _{\text {max}}$, high nutrient levels are consistently associated with larger magnitude and stronger amplification of nutrient fluctuations (Figs. 8c, 8d). Under nutrient-rich conditions, host growth reaches the threshold at which it is no longer nutrient-limited and transitions to a limit cycle at a smaller $\beta _{\text {max}}$ than under nutrient-poor conditions (Figs. 8g, 8h). The basic reproduction number ($R_0$) increases linearly with $\beta _{\text {max}}$, reaching about 6 at $N = 40$ g $N/m^2$ and more than doubling at $N = 80$ g $N/m^2$ (Figs. 8g, 8h). Together, these results show that nutrient availability modulates the magnitude of disease spread, as well as the sensitivity of the system to transmission parameters, effectively lowering the threshold for oscillatory dynamics.

**Fig. 9:**
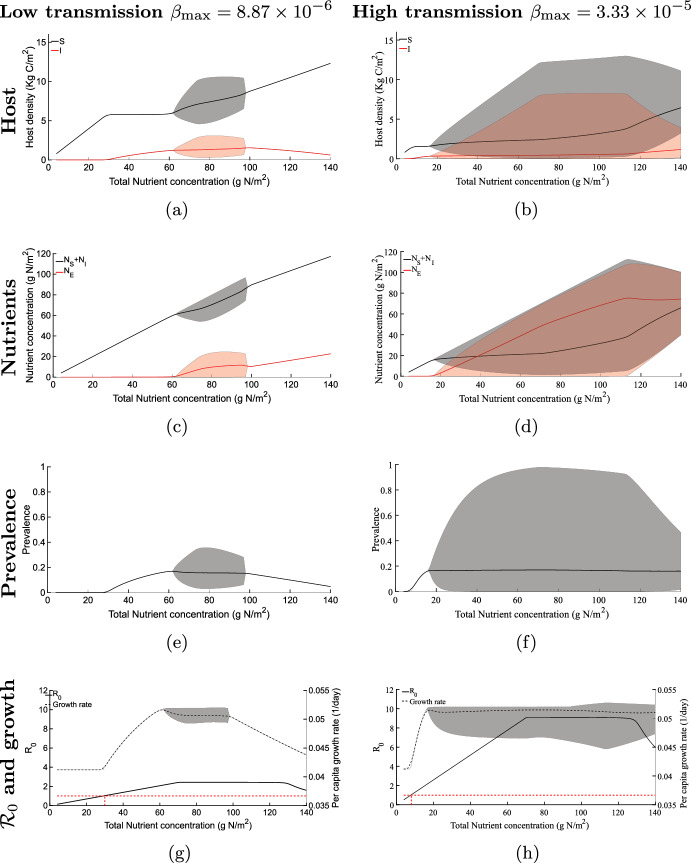
Bifurcation diagram of model (4) for varying values of *N* under two maximum transmission rates: (left) low transmission rate ($\beta _{\max }=8.87\times 10^{-6}$), and (right) high transmission rate ($\beta _{\max }=3.33\times 10^{-5}$), showing host density (a,b), host nutrient concentration (c, d), prevalence (e,f), basic reproduction number $\mathcal {R}_0$, and growth rate (g,h). Here $K=14$ kg $C/m^2$, and other parameter values are listed in Table 1. These bifurcation diagrams illustrate the long-term dynamics ($t > 20{,}000$ years), where curves show steady-state dynamics, shaded regions depict the maximum and minimum values of limit cycles, and curves inside shaded regions show average values of the limit cycles (color figure online)

Figure 9 shows carbon and nutrient fluxes among hosts (*S*, *I*, $N_S$, $N_I$) and abiotic nutrients ($N_E$) in model 4 across total nutrient concentrations (*N*) under low and high transmission rates. At low $\beta _{\text {max}}$, the disease persists for $N \ge 30$ g $N/m^2$. As *N* increases, the system undergoes a Hopf bifurcation near $N = 60$ g $N/m^2$. With further increases, the resulting limit cycles expand in amplitude, then weaken, and eventually collapse abruptly near $N = 95$ g $N/m^2$, giving rise to a new stable equilibrium (Fig. 9, first column). In contrast, at high $\beta _{\text {max}}$, the disease persists for $N \ge 10$ g $N/m^2$. As *N* increases, a Hopf bifurcation emerges near $N = 20$ g $N/m^2$. The limit cycles then expand in amplitude for $20 \le N \le 110$ before weakening at $N \ge 110$ (Fig. 9, second column). In summary, the maximum transmission rate ($\beta _{\text {max}}$) shifts the thresholds for disease persistence or extinction, the onset of Hopf bifurcations, the expansion and weakening of limit cycles, and the transition to stable equilibria. These results demonstrate that nutrient–disease feedbacks strongly shape the distribution of host densities and nutrients between autotrophs and the abiotic environment. This interaction highlights a key feedback mechanism: nutrient enrichment increases host biomass and transmission opportunities, while elevated transmission intensifies nutrient redistribution, jointly shifting dynamical thresholds.

#### Role of Infection on Uptake Efficiency in Disease-Nutrient Dynamics

**Fig. 10:**
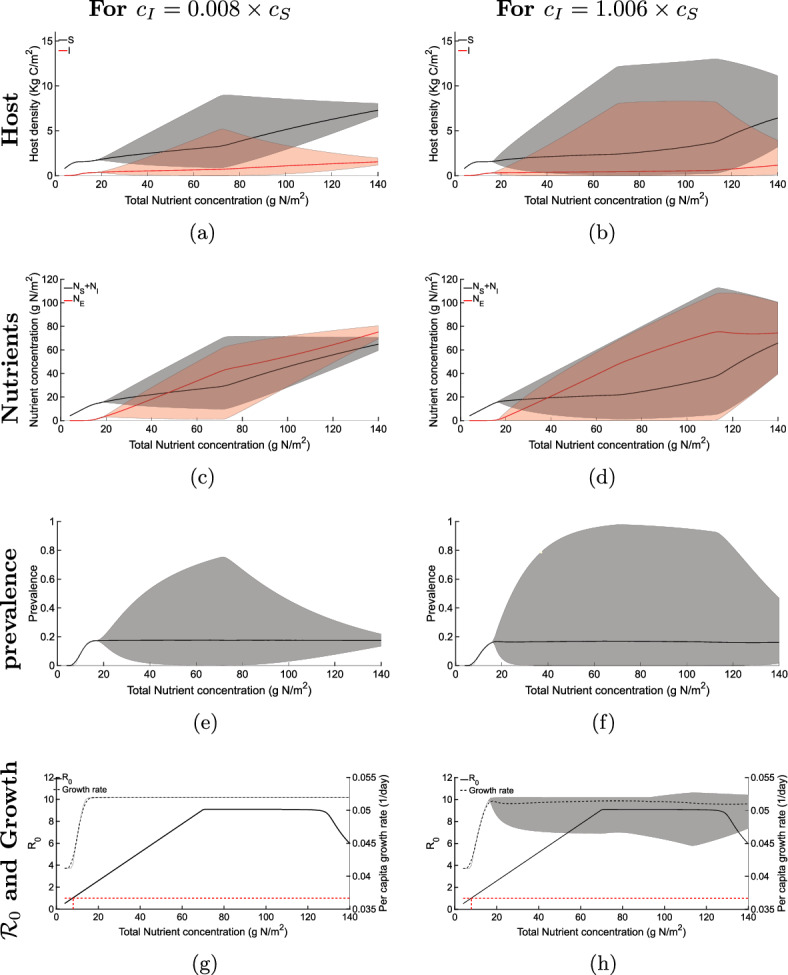
Bifurcation diagram of model (4) showing: Host density (1st row), Nutrient concentration in the host (2nd row), Prevalence (3rd row), and basic reproduction number $\mathcal {R}_0$ and growth rate (4th row) under C-dependent carrying capacity $K=14$ kg $C/m^2$. Other parameter values are listed in Table 1, and bifurcations are shown for varying values of *N* under two assumptions: (a) For $c_I=0.008 \times c_S$, and (b) For $c_I=1.006 \times c_S$. These bifurcation diagrams illustrate the long-term dynamics ($t > 20{,}000$ years), where curves show steady-state dynamics, shaded regions depict the maximum and minimum values of limit cycles, and curves inside shaded regions show average values of the limit cycles (color figure online)

Nutrient uptake and elemental composition frequently differ between infected and healthy tissues. Nutrient uptake can vary with infection status, as empirical studies show that infected and uninfected autotrophs often differ in their uptake capacity (Dordas 2008; Fones and Gurr 2017). Ecosystem models that fail to incorporate these infection-mediated differences in nutrient uptake risk missing key feedbacks in host–pathogen–nutrient interactions. We conducted numerical bifurcation analyses to investigate how system dynamics change when the nutrient uptake rate of infected hosts is either greater or smaller than that of susceptible hosts across a range of nutrient levels (*N*). When the uptake rate of infected hosts is lower ($c_I = 0.008 \times c_S$), both infected and susceptible hosts enter a limit cycle near $N = 20$ g $N/m^2$, with the amplitude increasing for $20 \le N \le 70$ and then weakening for $N > 70$ g $N/m^2$. In contrast, when the uptake rate is slightly higher ($c_I = 1.008 \times c_S$), both susceptible and infected hosts exhibit large-amplitude limit cycles that subsequently decline and weaken for $N > 110$ g $N/m^2$ (Figs. 10a, 10b). For $c_I = 0.008 \times c_S$, nutrient dynamics undergo a Hopf bifurcation near $N = 20$ g $N/m^2$, and the average environmental nutrient $N_E$ exceeds the host nutrient pool $N_S + N_I$ at $N = 50$ g $N/m^2$, remaining higher thereafter. With higher uptake ($c_I = 1.006 \times c_S$), this crossover occurs earlier, near $N = 40$ g $N/m^2$, and $N_E$ remains higher across the subsequent nutrient range (Figs. 10c, 10d). Across the full range of nutrient levels, when infected hosts have a higher uptake rate ($c_I = 1.006 \times c_S$), infection prevalence exhibits large, strongly amplified limit cycles, peaking near $N = 110$ g $N/m^2$, after which the oscillations decline and weaken. In contrast, when infected hosts have a lower uptake rate ($c_I = 0.008 \times c_S$), prevalence shows limit cycles that peak near $N = 70$ g $N/m^2$ and then decline and weaken thereafter (Figs. 10e, 10f). At $c_I = 0.008 \times c_S$, the long-term dynamics show minimal amplitude in oscillations of the growth rate, which increases and approaches a plateau under nutrient-rich conditions (Fig. 10g). In contrast, at $c_I = 1.006 \times c_S$, the growth rate exhibits wider amplitude oscillations across the nutrient gradient (Fig. 10h). Appendix 14 provides additional context for interpreting (Figs. 10g, 10h).

#### Two-Parameter Bifurcation (Resource disease feedback)

**Fig. 11:**
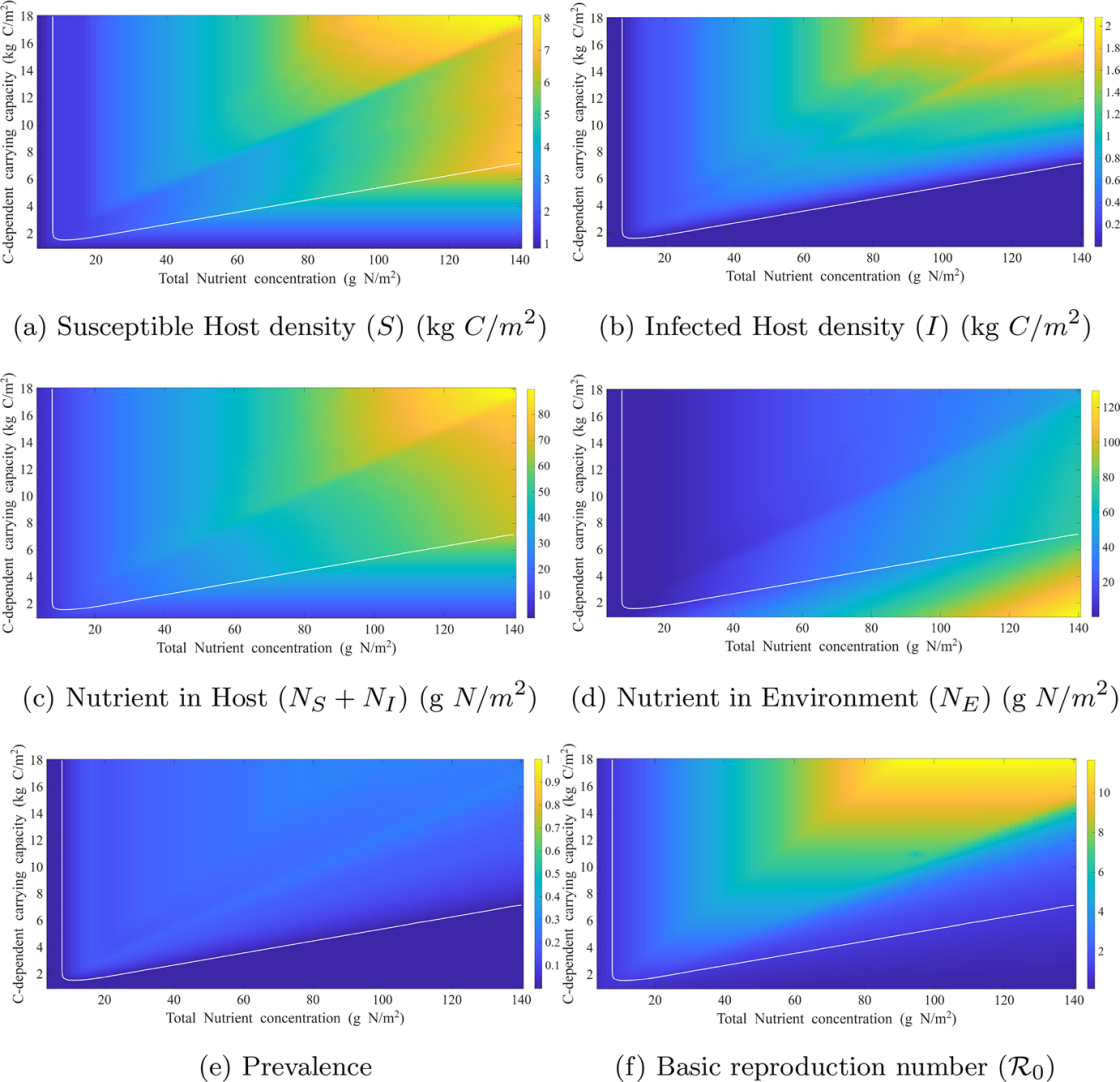
Two-parameter bifurcation diagram showcasing the long-term dynamics ($t>20,000$) years of (a) Susceptible host density (*S*), (b) Infected host density (*I*), (c) Nutrient in Host ($N_S+N_I$), (d) Nutrient in Environment ($N_E$), (e) Prevalence, and (f) Basic reproduction number ($\mathcal {R}_0$), for varying values of the *C*-dependent carrying capacity *K* (kg $C/m^2$) and total nutrient concentration *N* (g $N/m^2$) and other parameter values are listed in Table 1. For each (*N*, *K*) pair, trajectories were integrated beyond transient dynamics and show equilibria values, or when solutions approach stable limit cycles, values depict the mean over one full cycle. Colors, therefore, represent the asymptotic steady-state or mean periodic behavior of each variable. White contour lines indicate combinations of *K* and *N* where $\mathcal {R}_0 = 1$ (color figure online)

Plant density strongly influences interactions among plants by modifying physiological processes, plant architecture, canopy microclimate, and the dispersal and growth of pests and pathogens (Douma and Noordhoek 2025). Close proximity between host plants has long been proposed to enhance disease incidence by increasing the transmission of infectious propagules (Burdon and Chilvers 1976; Giesler et al. 1996). The influences of plant density and crowding can be captured through variations in the carbon-dependent carrying capacity (*K*). To broaden the perspective on resource–disease feedbacks, we explore the roles of *K* and total nutrient concentration (*N*) using two-parameter bifurcation analyses. These figures show the average host biomass, nutrient concentrations, infection prevalence, and basic reproduction number exhibited in long-term dynamics as heatmaps across *N* and *K* space. Shifts in *N* and *K* correspond to transitions in system dynamics, including movement from equilibria stability to Hopf bifurcations, the rise and collapse of limit cycles, and the abrupt emergence of stable equilibria across their ranges (Fig. 11). White contour lines indicate threshold combinations of the carbon-dependent carrying capacity (K) and total nutrient concentration (N) where the basic reproduction number ($\mathcal {R}_0$) equals one, separating regions of pathogen extinction ($\mathcal {R}_0 < 1$) and persistence ($\mathcal {R}_0 > 1$). The highest host biomass for both susceptible and infected hosts occurred when total *N* and *K* were high (Figs. 11a–11b). At lower values of *K* ($\textit{i.e.}, K < 3~kg~C/m^2)$), susceptible host density was minimal and remained unchanged with variation in *N*; at this point, host growth became light-limited. Likewise, when *N* was low ($\textit{i.e.}, N < 10~ g~N/m^2$), it remained unchanged with variation in *K*; at this point, host growth became Nutrient-limited (Fig. 11a). In Fig. 11b, the lower-right region enclosed by the white contour lines indicates infection extinction ($\mathcal {R}_0 < 1$). Here, the transmission rate is low due to high environmental nutrient concentration. Similarly, when *N* is low ($\textit{i.e.}, N < 10~g~N/m^2$), infection also vanishes because host density remains minimal across *K*, as host growth becomes nutrient-limited. Appendix 12 provides valuable insights for interpreting Figs. 11a–11b. Host nutrient concentration peaked at high *N* and *K* but remained low and unchanged at $K < 3$ kg $C/m^2$ or $N < 10$ g $N/m^2$ (Fig. 11c). Appendix 13 provides additional context for interpreting Fig. 11c. The highest environmental nutrient concentration was observed at low *K* ($\textit{i.e.}, K < 3$ kg $C/m^2$) and high *N* ($\textit{i.e.}, N > 120$ g $N/m^2$). The upper-left region of Fig. 11d shows nutrient-poor conditions, the central region moderate levels, and the lower-right region nutrient-rich conditions. At a low nutrient level ($N = 10$ g $N/m^2$), model dynamics approach a stable equilibrium with low infection prevalence, whereas at high nutrient levels, $\beta (N_E)$ decreases and infection prevalence declines toward extinction (Fig. 11e) (Fig. 11e). Changes in $\mathcal {R}_0$ primarily followed environmental nutrient levels ($N_E$) and susceptible host biomass, as $\beta (N_E)$ and *S* are key components in its calculation (15). The region enclosed by the white contour lines ($\mathcal {R}_0 < 1$) indicates infection extinction driven by nutrient-rich conditions or low host biomass, whereas the remaining parameter space represents combinations of *K* and *N* where infection persists ($\mathcal {R}_0 > 1$) (Fig. 11f).

## **Conclusion and Possible Future Explorations**

Disease and ecosystem nutrient dynamics are closely connected through multiple pathways. In this study, we use a disease-mediated nutrient dynamic framework and incorporate novel nutrient-driven transmission to capture the bidirectional nature of nutrient–disease interactions. A key contribution of this work is the introduction of a nutrient-dependent transmission function, $\beta (N_E)$, which directly links environmental nutrient availability to pathogen transmission within an established stoichiometric disease-nutrient framework. This framework reveals how feedback loops and emergent dynamics can arise, providing a bridge between disease ecology and ecosystem ecology. The reference model with constant transmission (model (1)) shows infection extinction at low nutrients, a delayed Hopf bifurcation near $N = 50$ g $N/m^2$, and stable limit cycles with $\mathcal {R}_0$ levels off around 3 under nutrient-rich conditions (Fig. 7, first column). In contrast, the nutrient-driven transmission model (4) exhibits extinction at lower nutrient levels, an earlier Hopf bifurcation, and transient limit cycles that initially expand and then weaken, with $\mathcal {R}_0$ peaking near 10 before declining (Fig. 7, second column). These results indicate that model (1) transitions into stable limit cycle dynamics that persist indefinitely once host growth is no longer nutrient-limited under high nutrient availability. In contrast, model (4) extends this framework by incorporating nutrient-driven transmission, $\beta (N_E)$, which increases its sensitivity to nutrient variation and generates stronger nonlinear feedbacks.

Bifurcation analyses across gradients of maximum transmission rate ($\beta _{\text {max}}$) under low and high nutrient levels show that nutrient enrichment lowers the threshold value of $\beta _{\text {max}}$ required for disease persistence (Fig. 8). The analyses further indicate that Hopf bifurcations and limit cycles emerge earlier and with greater amplitude under high nutrient availability. Similarly, bifurcation analyses across gradients of total nutrient concentration (*N*) under low and high $\beta _{\text {max}}$ show that higher transmission rates lower the nutrient threshold required for disease persistence and shift the onset of oscillatory dynamics to lower nutrient levels (Fig. 9). These results arise because nutrient enrichment increases host biomass, thereby enhancing opportunities for transmission even at lower values of $\beta _{\text {max}}$. At the same time, higher transmission rates intensify infection pressure, enabling disease persistence under reduced nutrient availability. Collectively, these results demonstrate that nutrient–disease feedbacks play a central role in shaping the distribution of host densities and nutrients between autotrophs and the abiotic environment.

Empirical evidence shows that infected and susceptible autotrophs often differ in nutrient uptake capacity (Dordas 2008; Fones and Gurr 2017), and our bifurcation results demonstrate that even small differences in infected host uptake rates can strongly alter system dynamics. A lower uptake rate dampens the amplitude of limit cycles in host densities, nutrient concentrations, and prevalence, while the growth rate converges without oscillations, leading to weaker and eventually stable dynamics. In contrast, a higher uptake rate amplifies nonlinear feedbacks, generating large and persistent oscillations in host densities, nutrient pools, prevalence, and growth (Fig. 10). These patterns arise because higher uptake stimulates the bottom-up influence of environmental nutrient availability on disease dynamics (Aalto et al. 2015; Borer et al. 2016; Civitello et al. 2018; Dordas 2008), while simultaneously reinforcing the top-down feedback of disease on ecosystem nutrient cycling (Eviner and Likens 2008; Fischhoff et al. 2020; Preston et al. 2016).

Two-parameter bifurcation analyses across total nutrient supply (*N*) and carbon-dependent carrying capacity (*K*) reveal thresholds that shape host–pathogen–nutrient dynamics (Fig. 11). Host biomass and nutrient concentrations peak when both *N* and *K* are high, but remain minimal at low *K* or low *N* as growth becomes light-limited at low *K* and nutrient-limited at low *N*. Variation in *N* and *K* drives transitions from stability to Hopf bifurcations, the rise and collapse of limit cycles, and the abrupt emergence of stable equilibria. Environmental nutrients ($N_E$) are greatest under low *K* and high *N*. The region enclosed by the white contour lines ($\mathcal {R}_0 < 1$) indicates infection extinction driven by nutrient-rich conditions or low host biomass, whereas the remaining parameter space represents combinations of *K* and *N* where infection persists ($\mathcal {R}_0 > 1$). Overall, our results underscore the importance of incorporating resource-dependent transmission mechanisms when seeking to understand and predict disease dynamics in nutrient-limited ecosystems.

Future work should extend the present nutrient-driven transmission framework to incorporate multiple transmission pathways, capturing both indirect nutrient-mediated effects and direct vector-borne transmission, particularly via nitidulid beetles responsible for the spread of oak wilt (*Bretziella fagacearum*) (Juzwik et al. 2011). Integrating an explicit vector component within spatially structured patch or metapopulation models would enable investigation of how landscape connectivity, host distribution, and vector dispersal jointly regulate invasion thresholds and disease persistence. Such spatial-vector coupling is especially important for forest systems, where local root graft transmission and long-distance insect-mediated dispersal interact across heterogeneous environments.

In addition, incorporating microbes in the nutrient decomposition processes into the nutrient-driven transmission framework would provide a more mechanistic representation of nutrient cycling and its feedback on host susceptibility and pathogen dynamics, consistent with recent advances in stoichiometric disease ecology (Borer et al. 2022). These extensions would enable the development of a unified, multi-scale modeling framework that integrates nutrient dynamics, vector ecology, and spatial structure, thereby enhancing the ecological realism and improving the predictive capability of disease-ecosystem models. Moreover, integrating such developments with data-driven parameterization and sensitivity analyses would strengthen the applicability of the model for understanding and managing forest diseases such as oak wilt.

## Figures and Tables

**Fig. 12:**
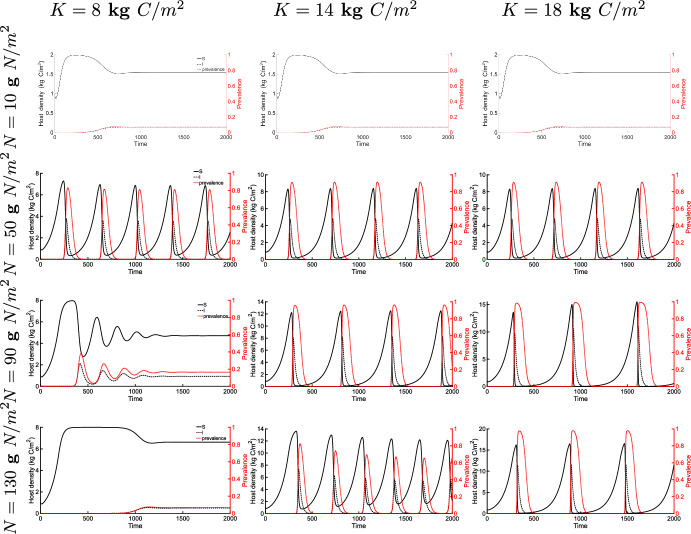
Numerical simulation of the model (4) showing host density and prevalence under varying values of total nutrient concentration *N* and C-dependent carrying capacity *K*, using parameter values listed in Table 1. These simulations provide important context for interpreting the bifurcation diagrams of model (4) under different nutrient conditions (color figure online)

**Fig. 13:**
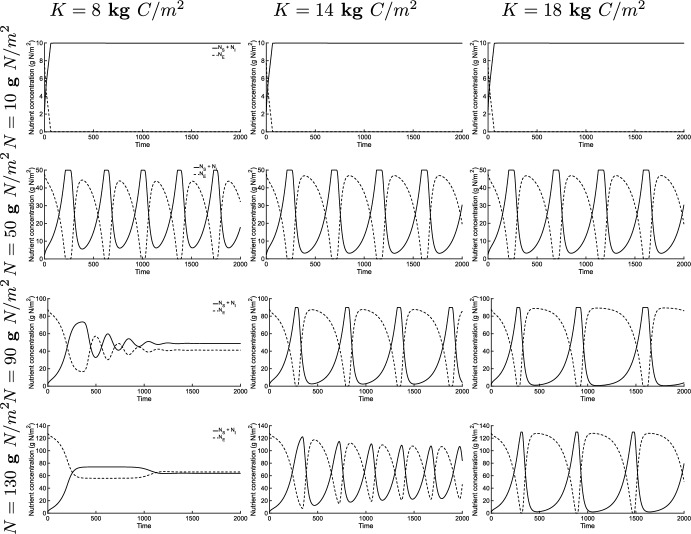
Numerical simulation of the model (4) showing nutrient concentrations in autotrophs ($N_S+N_I$) and the environment ($N_S$) under varying values of total nutrient concentration *N* and C-dependent carrying capacity *K*, using parameter values listed in Table 1. These simulations provide important context for interpreting the bifurcation diagrams of model (4) under different nutrient conditions (color figure online)

**Fig. 14:**
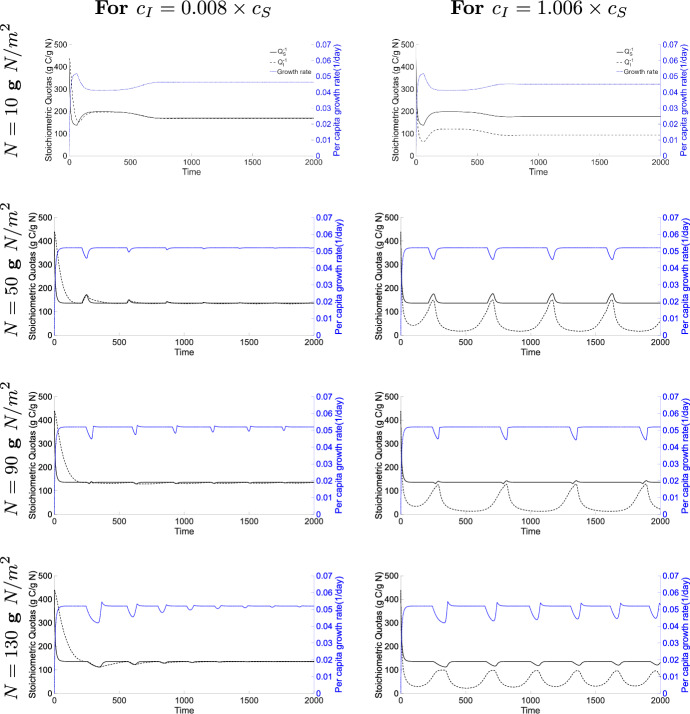
Numerical simulations of the model (4) show the growth rate and stoichiometric quota under varying values of total nutrient concentration *N* and infected host uptake Rate ($c_I$), using parameter values listed in Table 1. These simulations provide important context for interpreting the bifurcation diagrams of model (4) under different nutrient conditions and uptake rates that vary with the health status of the host (color figure online)

**Fig. 15:**
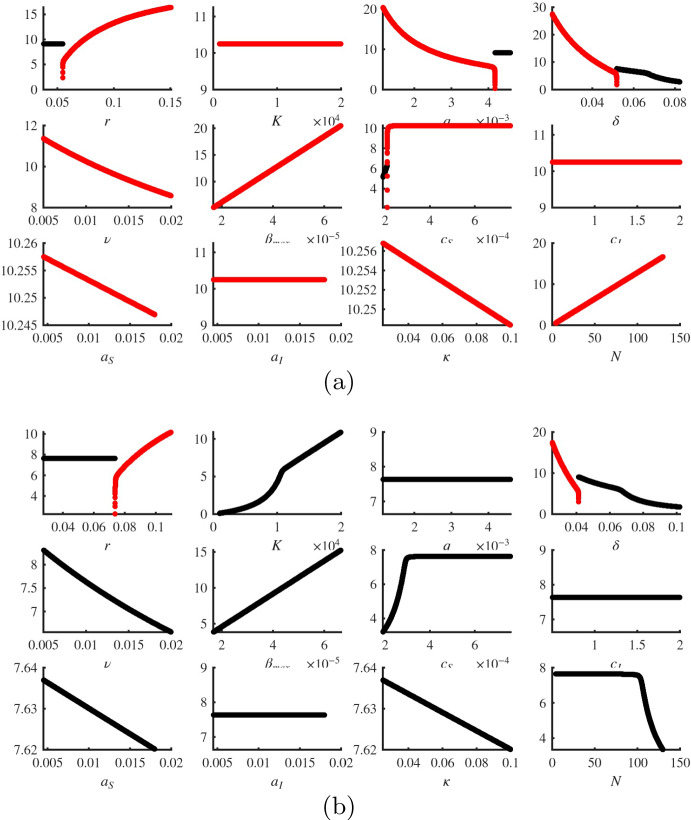
Monotonicity plots for the basic reproduction number $\mathcal {R}_0$ evaluated at the disease-free equilibria $E_1$ or $E_2$. (a) $\mathcal {R}_0$ computed primarily at $E_2$ under parameter ranges from half to twice their baseline values (Table 1). (b) $\mathcal {R}_0$ computed primarily at $E_1$ under the same parameter ranges, except with baseline values fixed at $r=0.055$ and $\delta =0.051$. In the plots, black points correspond to parameter values for which $\mathcal {R}_0$ is evaluated at $E_1$, whereas red points correspond to parameter values for which $\mathcal {R}_0$ is evaluated at $E_2$. By adjusting the baseline values of *r* and $\delta$, the plots can be obtained with $\mathcal {R}_0$ evaluated entirely at either $E_1$ or $E_2$ (color figure online)

## Additional Numerical Simulations

### Simulations of Model Under Varying Total Nutrient Concentrations (*N*) and Different Carbon-Dependent Carrying Capacities (*K*)

Bifurcation analyses show that variation in *N*-*K* space impacts whether host–pathogen–nutrient dynamics settle into equilibrium, undergo oscillations, or collapse to extinction. Numerical simulations of the model (4), illustrating host population density, infection prevalence, and the distribution of nutrients between autotrophs and the environment under varying total nutrient concentrations (*N*) and different values of carbon-dependent carrying capacity (*K*), are presented here. These simulations provide valuable insights for interpreting the model’s bifurcation diagrams.

### Numerical Simulations Under Varying Total Nutrient Concentrations (*N*) and Infected Host Uptake Rates

Numerical simulations of model (4), illustrating host stoichiometry and growth rate under varying total nutrient concentrations (*N*) and two different values of the nutrient uptake rate of the infected host, are presented here. These simulations provide valuable insights for interpreting the model’s bifurcation diagrams (Fig. 10)

## Latin Hypercube and Partial Rank Correlation Coefficient

The relationships between model parameters and the basic reproduction number $\mathcal {R}_0$, evaluated at the disease-free equilibrium $E_1$ or $E_2$ that exists under the corresponding parameter values, are shown in Figs. (15a, 15b). In Fig. 15a, $\mathcal {R}_0$ is computed primarily at $E_2$ under parameter ranges from half to twice their baseline values (Table 1). In Fig. 15b, $\mathcal {R}_0$ is computed primarily at $E_1$ under the same parameter ranges, except with baseline values fixed at $r=0.055$ and $\delta =0.051$. For most parameters, $\mathcal {R}_0$ varies monotonically, showing a consistent increase or decrease across the parameter range, except in regions where the disease-free equilibrium switches between $E_1$ and $E_2$. Overall, these results indicate that monotonic relationships between individual parameters and the basic reproduction number $\mathcal {R}_0$ hold at the corresponding disease-free equilibrium ($E_1$ or $E_2$) over the ranges considered. Therefore, the PRCC analysis was performed using parameter ranges from half to twice their baseline values (Table 1), as supported by the monotonicity plot shown in Fig. 15a.

## Feasibility Of Equilibria

One of the disease-free equilibrium of system (4),

$$\begin{aligned} \left( \overline{S}, 0, \overline{N_S}, 0, \overline{N_E}\right) = \left( K,\,0,\, \frac{\phi + \sqrt{\phi ^2-4\delta c_s K N}}{2\delta },\,0,\, N-\frac{\phi + \sqrt{\phi ^2-4\delta c_s K N}}{2\delta } \right) , \end{aligned}$$

where

$$\begin{aligned} \phi =c_sK+\delta (N+a_s)>0, \qquad \text {and} \qquad \phi ^2 \ge 4\delta c_s K N, \end{aligned}$$

is not biologically feasible because $\overline{N_E}<0$.

### Proof

Set

$$\begin{aligned} B=\delta N-c_sK-\delta a_s. \end{aligned}$$

Then

$$\begin{aligned} \phi ^2-4\delta c_sKN =(c_sK+\delta (N+a_s))^2-4\delta c_sKN =B^2+4\delta ^2Na_s. \end{aligned}$$

Hence

$$\begin{aligned} N-\frac{\phi +\sqrt{\phi ^2-4\delta c_sKN}}{2\delta } = \frac{B-\sqrt{B^2+4\delta ^2Na_s}}{2\delta }. \end{aligned}$$

Since $\delta >0$, $N>0$, and $a_s>0$, we have

$$\begin{aligned} \sqrt{B^2+4\delta ^2Na_s}>|B|. \end{aligned}$$

Therefore

$$\begin{aligned} B-\sqrt{B^2+4\delta ^2Na_s}<0, \end{aligned}$$

and because $2\delta >0$, it follows that

$$\begin{aligned} N-\frac{\phi +\sqrt{\phi ^2-4\delta c_sKN}}{2\delta }<0. \end{aligned}$$

Thus, it is not biologically feasible because $\overline{N_E}<0$. $\square$
